# Establishment and application of a nomogram diagram for predicting calcium oxalate stones in patients with urinary tract stones

**DOI:** 10.1007/s00240-024-01542-x

**Published:** 2024-03-01

**Authors:** Guanhua Zhu, Lichen Jin, Yinsheng Guo, Lu Sun, Shiqing Li, Feng Zhou

**Affiliations:** Department of Urology, The First Affiliated Hospital of Soochow University, 188 Shizi Street, 899 Pinghai Road, Soochow, 215006 Jiangsu China

**Keywords:** Morpho-constitutional analysis of urinary stones, Nomogram diagram, Calcium oxalate, Urinary ionic composition, Kidney stones

## Abstract

This retrospective study aims to examine the correlation between calcium oxalate (CaOx) stones and common clinical tests, as well as urine ionic composition. Additionally, we aim to develop and implement a personalized model to assess the accuracy and feasibility of using charts to predict calcium oxalate stones in patients with urinary tract stones. A retrospective analysis was conducted on data from 960 patients who underwent surgery for urinary stones at the First Affiliated Hospital of Soochow University from January 1, 2010, to December 31, 2022. Among these patients, 447 were selected for further analysis based on screening criteria. Multivariate logistic regression analysis was then performed to identify the best predictive features for calcium oxalate stones from the clinical data of the selected patients. A prediction model was developed using these features and presented in the form of a nomogram graph. The performance of the prediction model was assessed using the C-index, calibration curve, and decision curve, which evaluated its discriminative power, calibration, and clinical utility, respectively. The nomogram diagram prediction model developed in this study is effective in predicting calcium oxalate stones which is helpful in screening and early identification of high-risk patients with calcium oxalate urinary tract stones, and may be a guide for urologists in making clinical treatment decisions.

## Introduction

Updated data from the National Health and Nutrition Examination Survey (NHANES) shows a further increase in prevalence to 9.4% in women and 11.9% in men in the 2017–2018 cycle. Similarly, the annual healthcare burden of urolithiasis has increased significantly and is projected to exceed $4 billion by 2030 in the United States. The prevalence of urolithiasis varies worldwide, with estimates ranging from 7 to 13% in North America, 5–9% in Europe, and 1–5% in Asia. These variations are attributed to sociodemographic, lifestyle, environmental, genetic, and economic factors [1]. Stone production in the excretory system, particularly in the kidneys, ureters, and bladder, is primarily due to the buildup of organic compounds and minerals. This phenomenon can be influenced by a range of factors such as genetic predisposition, environmental conditions, and dietary habits [2]. China is one of the world's top three stone-prone regions. According to the latest epidemiological studies, the prevalence of urinary stones is high among Chinese adults, with a prevalence rate of 5.8%, including 6.5% for men and 5.1% for women. The prevalence of kidney stones among Chinese adults is 5.8%, with a prevalence of 6.5% among men and 5.1% among women. Almost 1 in 17 people suffer from kidney stones and the prevalence is higher in the south than in the north, and higher in rural than in urban areas [3]. Of these patients, 25% require hospitalization. Despite the marked improvement in stone clearance rates to date, there is still a high rate of stone recurrence, with a recurrence rate of up to 50% within 10 years [4]. Therefore, how to effectively prevent recurrence of urinary stones after treatment has become the focus of attention in urology. Meanwhile, calcium oxalate is the most common component of urinary stones, accounting for about 80%, so the study on the prediction of calcium oxalate stones is of great clinical significance. At this stage, it is believed that factors affecting stone composition include age, gender, race, drug use, genetics, dietary intake, environmental factors, insulin resistance and drinking water content [5–8]. It has been shown that CaOx stone formation is associated with features of the metabolic syndrome (MS), including elevated blood pressure, dyslipidemia, obesity, and glucose intolerance [9–12].

Predictive modeling by deep learning in computers to predict stone composition has had a continuous upward trend in recent years, but many primary hospitals do not have these conditions and their applicability is still very limited. Therefore, we tried a new method of column line graphs to predict urinary tract stone composition in treated patients.

## Information and methods

The data from the First Affiliated Hospital of Soochow University comprises information on 478 patients who underwent surgical treatment for urinary stones from January 1, 2010, to December 31, 2022. The exclusion criteria were [1] individuals with incomplete preoperative clinical data and [2] those with postoperative specimens that were not analyzed for stone composition. Among these patients, there were 322 males and 156 females. Out of the total cases, 353 patients had calcium oxalate stones, while the remaining 124 cases involved other types of stones. Stone samples were collected using methods such as percutaneous nephrolithotomy and ureteral rigid/flexible endoscopy. These samples were then analyzed for stone components at the hospital using infrared spectroscopy. Additionally, blood samples were obtained from fasting patients who had abstained from eating for at least 8 h overnight. The patient’s morning urine is usually collected, with a predominance of mid-stream urine.

## Diagnostic criteria

Abnormal glucose metabolism: according to the classification criteria of glucose metabolic status established by the WHO Expert Committee on Diabetes Mellitus based on different blood glucose criteria.

Hypercalciuria: for a 24 h urine collected during a regular diet hypercalciuria is defined as a calcium excretion greater than 4 mg/kg body weight, respectively as greater than 300 mg/24 h for men and 250 mg/24 h for women.

Hyperuricemia: in a normal purine dietary state, non-same-day twice fasting blood uric acid level is higher than 420 μmol/L for men and 360 μmol/L for women.

Hyperlipidemia: 1. hypercholesterolemia: total cholesterol level is higher than 5.7 mmol/L; 2. hypertriglyceridemia: triglyceride level is higher than 1.7 mmol/L; 3. mixed hyperlipidemia: triglyceride level is higher than 1.7 mmol/L, LDL cholesterol level is higher than 3.37 mmol/L; 4. low-density lipoproteinemia: HDL is lower than 1.04 mmol/L; 5. low-density lipoprotein anemia: HDL is lower than 1.04 mmol/L; 6. low-density lipoprotein anemia: HDL is lower than 1.04 mmol/L. 1.04 mmol/L.

Classification of stone components: The Mayo Clinic Stone Classification Practice and the European Urological Association guidelines classify stones. A stone is classified as calcium oxalate (CaOx) if either type of calcium oxalate (calcium oxalate monohydrate or calcium oxalate dihydrate) makes up more than 50% of the stone. Stones were classified as calcium phosphate (CaP) stones if they contained a majority (50%) of carbon apatite, or if they contained tricalcium phosphate, calcium dihydrogen phosphate dihydrate (DCPD) or amorphous calcium phosphate. Stones were classified as uric acid stones if they contained > 50% uric acid. Stones containing > 10% struvite, was classified in the infected stone group. Similarly, stones containing any cystine were categorized in the cystine group.

## Exclusion criteria

Pregnant or breastfeeding women and pediatric patients under 18 years of age, patients with renal tubular acidosis, patients with abnormal parathyroid function, cirrhosis, etc. and congenital anomalies of the urinary system, etc., hepatic insufficiency, patients taking medications (e.g., uric acid-lowering drugs, lipid-lowering drugs) or with the presence of other observational markers that could influence the inclusion of the present study, and patients with incomplete clinical data.

## Collection of clinical information

Clinical data of patients who met the inclusion and exclusion criteria were searched and obtained through the hospital's inpatient comprehensive query system, which collected basic information including patients' gender, age, height, past medical history and blood pressure status, stone composition and stone location. In addition, the patients' laboratory tests were also collected, including urine pH, urine specific gravity, urea nitrogen, creatinine, uric acid, blood lipid levels (triglyceride, total cholesterol, high/low density lipoprotein), blood electrolyte levels (blood calcium, blood phosphorus), 24 h urinalysis, and analysis of urinary ionic composition (ionized magnesium Mg, ionized calcium Ca, oxalate ion C_2_O_4_, phosphate ion PO_4_, citrate ion C_6_H_6_O_7_), etc.

## Statistical methods

Data processing in this experiment utilized SPSS version 25 for analysis and R version 4.3.0 software. Prism version 8.0 was employed to plot the Receiver Operating Characteristic (ROC) curve. The independent variables with a significance level (*P* < 0.1) in the univariate logistic regression analysis were included in the multifactorial logistic regression analysis. The Mann *G*–Whitney *U*-test was utilized to calculate the *P*-value, and Spearman correlation analysis was conducted for correlation analysis. A significance level of *P* < 0.05 was considered statistically significant. Consequently, independent risk factors and predictive factors of urinary calcium oxalate calculus were identified. The model’s differentiation and calibration were evaluated and validated using two indicators. The model’s differentiation ability was evaluated using ROC and Area under the Curve of ROC (AUC), while the calibration ability was assessed using the Hosmer–Lemeshow test and the calibration curve.

## Selection of sample size

According to the international norms of probabilistic predictive modeling, the number of patients in the dataset who develop calcium oxalate stones should satisfy more than 10 times the number of independent variables in the model [13]. In the prediction model constructed in this study, there are a total of six independent variables, and to ensure the reliability and stability of this model, the number of cases of patients who developed calcium oxalate stones required in the dataset should be at least 60 cases. Based on the incidence of calcium oxalate stones in this study, the required sample size of the dataset should be at least 180 cases. The number of cases included in the dataset of this study meets the above guidelines.

## Results

### Predictive modeling of calcium oxalate stones

**Fig. 1:**
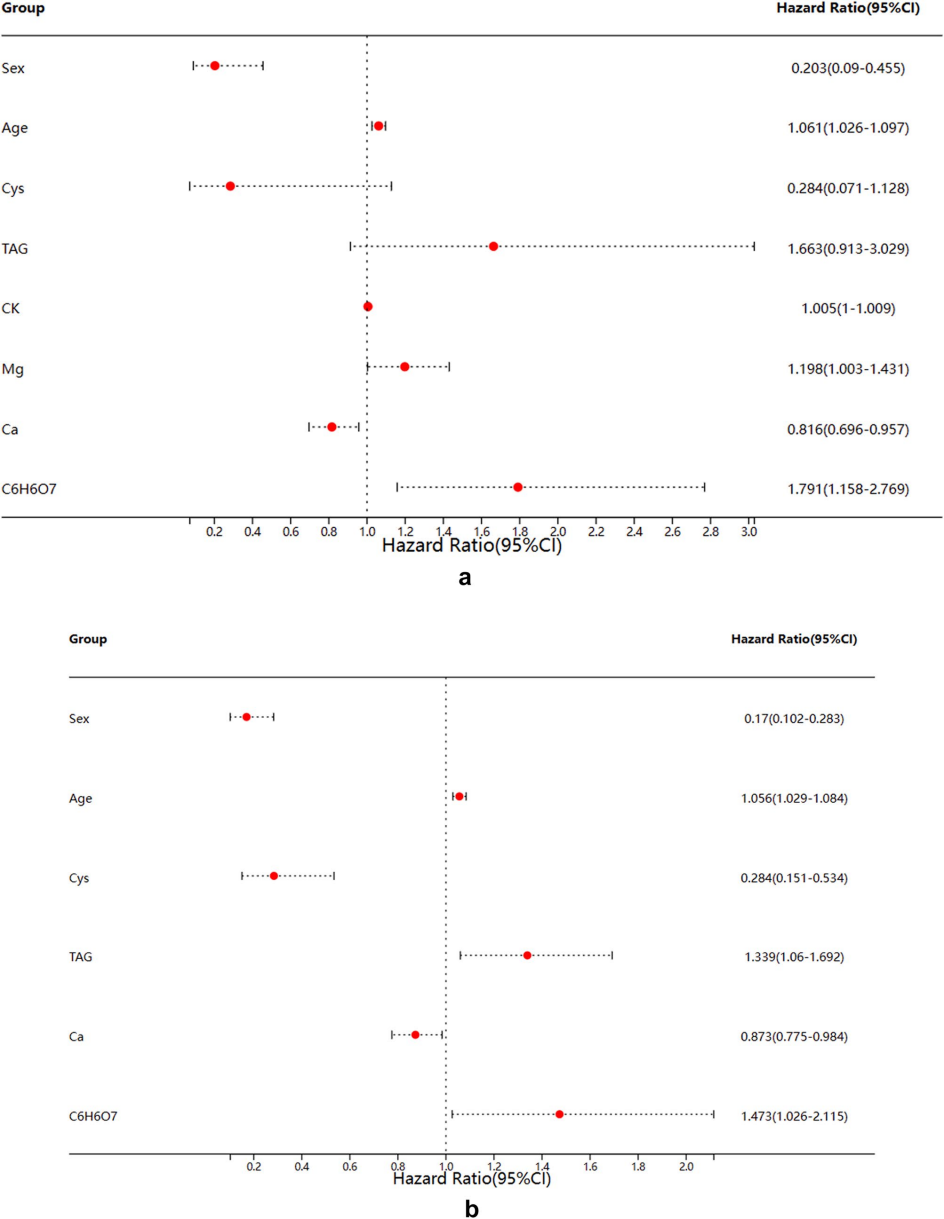
Forest plots for univariate analysis logistic regression analysis and multivariate logistic regression

Six independent factors affecting stone composition were screened by univariate and multifactorial logistic regression analysis (Fig. 1). The regression equation for this analysis was established: ln(*p*/1−*p*) = −0.367−1.771* Sex (1 for males and 2 for females) + 0.055* Age−1.258* Cystatin C + 0.292* Triglycerides—0.135* Ionic Calcium + 0.387* C_6_H_6_O_7_ (where *p* represents the probability that the stone composition is calcium oxalate and 1−p represents the probability that the stone composition is other stones).

### Visualization of nomogram creation

**Fig. 2:**
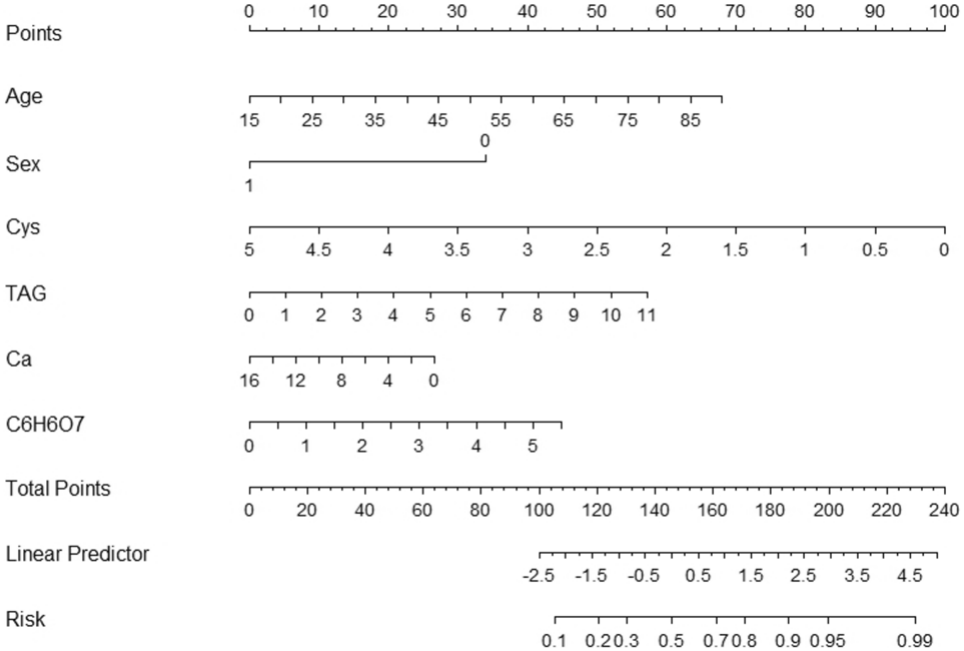
Nomogram chart

The best predictive features selected in the logistic regression analysis included gender, age, cystatin C, triglyceride, ionized calcium, and citrate ion C_6_H_6_O_7_. After the above best predictive features were modeled as a nomogram graph (Fig. 2).

The specific application of the DCA curve can be seen in this example below: if there is a patient with stones, female, 70 years old, with a Cys (cystatin C) of 3, TAG (triglyceride) of 11, Ca (ionized calcium) of 10, and C_6_H_6_O_7_ of 3, which corresponds to a total score of 0 + 50 + 40 + 55 + 10 + 23 = 178, the risk index for her stones to be calcium oxalate stones would be 0.81, which is 81 percent.

**Fig. 3:**
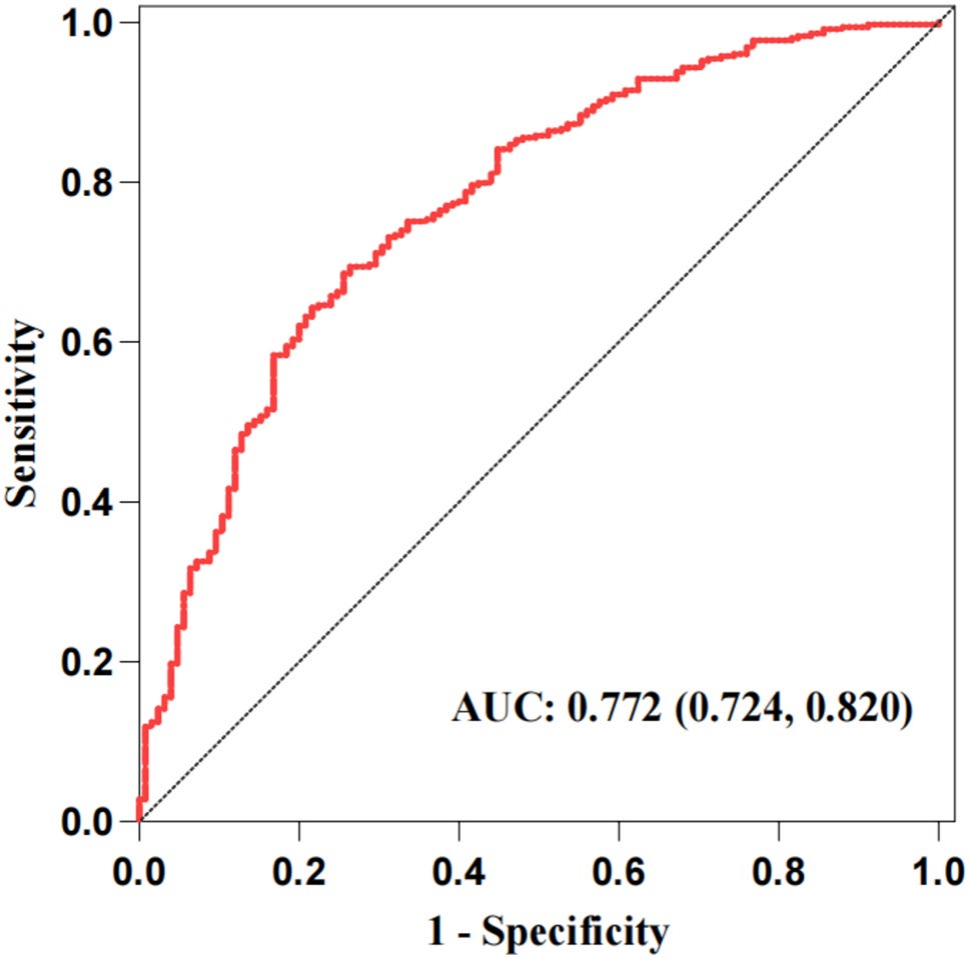
ROC curve

**Fig. 4:**
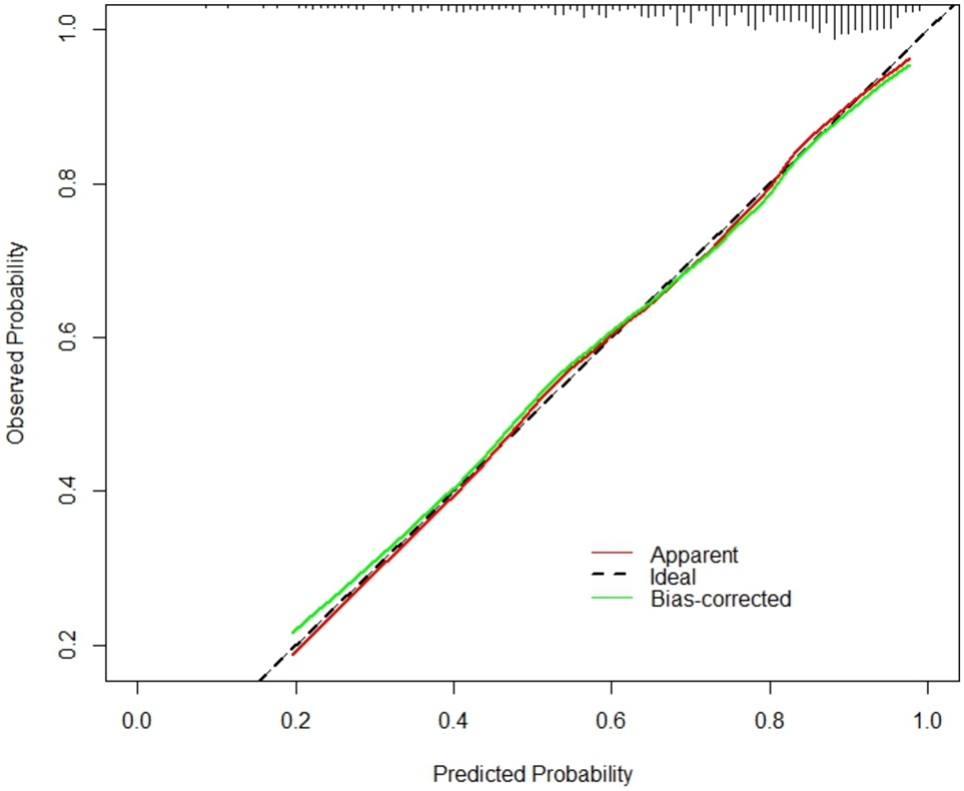
Calibration curves for calcium oxalate stone risk prediction models (*N* = 478, mean absolute error = 0.009)

**Fig. 5:**
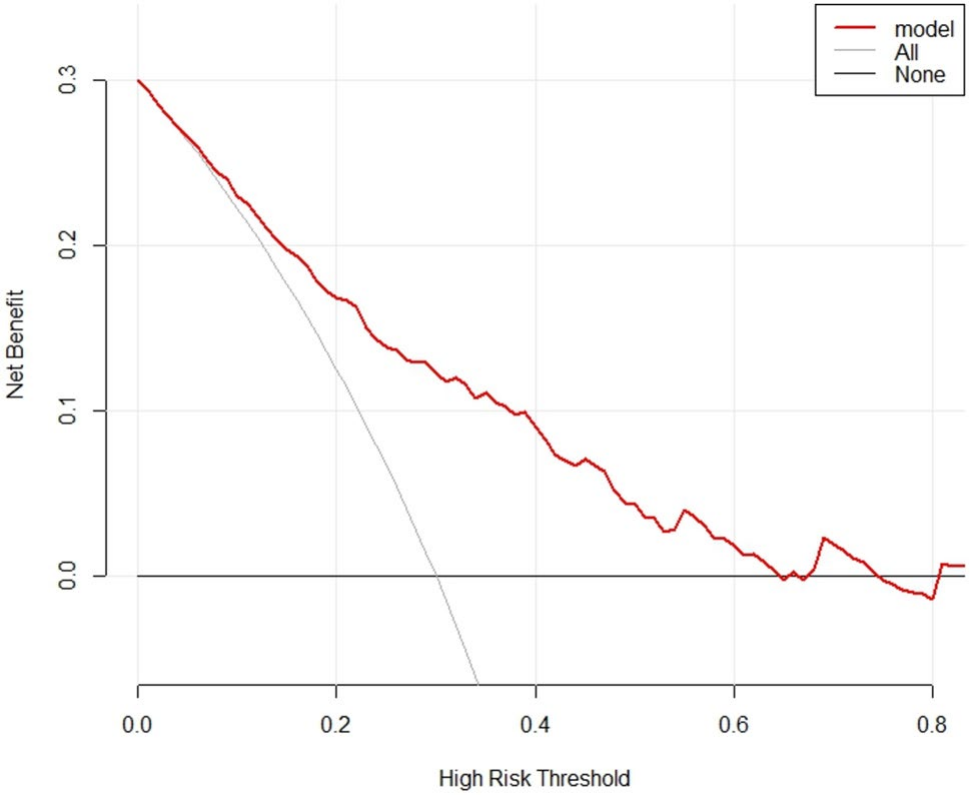
DAC curve

The AUC value and concordance index (C-index) were 0.772 and 0.7721, respectively (Fig. 3), which indicated that the model had good discriminative ability. The standard curve in the calibration curve fits well with the prediction calibration curve, suggesting a good calibration effect (Fig. 4). Decision Curve Analysis (DCA) decision curve analysis results showed that when the probability of the patient was 8–77%, the Nomogram of this study was used to predict the probability of calcium oxalate calculus and the corresponding diagnosis and treatment measures were given to the patient. Compared with medical intervention for all patients or no medical intervention for all patients, the net clinical benefit would be higher (Fig. 5).

## Discussions

In urolithiasis, calcium-containing stones predominate, mainly including calcium oxalate stones and calcium phosphate stones, accounting for more than 70% of all urinary stones; the rest also include uric acid stones, magnesium ammonium phosphate stones, cystine stones and mixed stones, etc. [14]. Previous studies have concluded that knowledge of stone composition is fundamental to the prevention and treatment of urinary tract stones.

Early studies have found that stones can be categorized on the basis of urography (KUB) and computed tomography (CT) imaging findings, varying according to mineral composition [15]. However, this is a rough and vague categorization and the accuracy of the results needs to be improved. The ability of electron computed tomography (CT) to predict stone composition has been studied for decades, and some controversy still exists. Some studies have shown that monoenergetic and dual-energetic computed tomography (CT) possess better discriminatory ability [16]; Takashi Kawahara et al. found that non-contrast computed tomography improved the predictive value of differentiating stone composition from Heinz units (Hu) obtained from CT scans. The HUs in calcium oxalate were significantly higher than those in uric acid (*p* < 0.01) and struvite (*p* < 0.01). Those in monohydrate stones were significantly higher, compared with dehydrate stones (*p* < 0.05). However, a number of factors also tend to influence Hu measurements on non-enhanced computed tomography (NCCT), such as stone size and mixed stone composition, making the prediction inaccurate [17]. Weronika Sofińska-Chmiel et al. demonstrate the utilization of infrared spectroscopy (FTIR), X-ray diffraction (XRD), and electron microscopy with an EDX detector to identify components associated with urinary stones [18]. as well as the rising trend in recent years of building predictive models through deep learning in computers to predict stone composition [19, 20], Many primary care hospitals do not have these conditions and its applicability is still very limited. So we tried a new method, column line drawing, to predict calcium oxalate stones in patients with pre-treatment urinary tract stones.

In this study, compared to the traditional serum and 24 h urine tests [21], We conducted a study analyzing the urinary ionic composition of patients pre-surgery, investigating factors such as gender, age, cystatin C, triglycerides, creatine kinase, ionized calcium Ca, ionized magnesium Mg, and citrate ion C_6_H_6_O_7_ that may influence the results. Several nomograms based on urinary calcium, oxalate citrate, and magnesium have been developed in the past by previous authors. However, we have utilized the latest statistical methods relative to previous studies, while adding new variables. This graph effectively distinguishes calcium oxalate stones from other types. The nomogram can help clinicians identify patients at high risk of calcium oxalate stones early on, and involves patients in the diagnosis and treatment decision-making process. Additionally, the nomogram graph can be used in both outpatient and inpatient settings, benefiting resource-limited rural areas in China. It also serves as a simple and effective diagnostic and treatment tool for patients unable to undergo surgery (Fig. 5).

First, similar to previous studies, men were associated with a higher risk, suggesting that men were more likely to develop calcium oxalate stones [22]. This may be related to the nature of men’s occupation: men tend to perform more physical labor and sweat more, leading to supersaturation and precipitation of crystals in the urine. Ahmed M Elshal et al. found that the expression of ARs and miRNA-185-5p was significantly higher in SFGs than in the healthy population by comparing 74 patients with CaOx stones (SFGs) with 40 healthy individuals. However, CSF-1 expression was significantly lower in the stone group than in the healthy subjects. Wei Zhu et al. showed that inhibition of androgen receptor (AR) expression in renal tubular epithelial cells increased macrophage recruitment/M2 polarization, which resulted in enhanced phagocytosis of CaOx crystals in the kidney, confirming that estrogens inhibit the formation of stones more than androgens do in the human body [23, 24].

Secondly, age is another important factor in the formation of urinary stones, especially calcium-containing stones, and the reasons for this are still controversial. Perinpam et al. showed that calcium excretion declines with age [25]. However, the cohort evaluated in this study consisted primarily of non-stone-formers. Triet Vincent M. Tran et al. concluded that calcium excretion in patients with CaOx stones was significantly reduced with decreasing creatinine clearance (CrCl), with a generalized relationship to advancing age [26]. Some studies suggest that stones can take decades to develop. This immediately implies that the stone burden will increase with age.

In addition, this study found that cystatin C, triglycerides, ionized calcium Ca, and citrate ion C_6_H_6_O_7_ could also be important factors as new predictors of calcium oxalate stones.

Mao et al. [27] showed that cystatin C was positively correlated with the degree of hydronephrosis caused by kidney stones. Hyperlipidemia is associated with decreased urinary pH and increased urate excretion, and acidic urine may lead to uric acid stone formation. Elevated uric acid excretion is a risk factor not only for uric acid stone formation but also for CaOx stone formation [28, 29]. Stone formation occurs due to the urine being oversaturated with calcium oxalate and brushite. The level of oversaturation is linked to fluid intake and the quantity of citrate and calcium in the urine. Urinary calcium levels exceeding 200 mg/day elevate the risk of stones and frequently result in a negative calcium balance. Reduced calcium reabsorption by the kidneys is a factor in idiopathic hypercalciuria [30].

## Conclusion

We preliminarily screened five factors that may influence the composition of urinary stones in patients by statistical analysis and obtained certification of previous studies. However, this study has limitations. Firstly, although we set strict inclusion and exclusion criteria to reflect real disease occurrence, there may still be a possibility of selection bias in a retrospective study. Secondly, the prediction model's data are relatively insufficient and comes from a single center with a small sample size. It has not been tested on patients with urolithiasis admitted to other centers or medical institutions, so further verification is needed to assess its applicability. Thirdly, the study only considered dichotomous variables for outcome indicators, neglecting mixed stones and simplifying the model slightly. Finally, due to incomplete information, other important disease parameters could not be analyzed. To improve accuracy, better data sources and adjustments to the column line graphs are necessary. In this study, we developed a personalized nomogram diagram prediction model for calcium oxalate stones in patients with urinary tract stones. This model is simple, quick, and visually appealing, providing a comprehensive overview of the prediction model. It demonstrates good discriminatory ability, which can enhance early identification and treatment for these patients.

## Data Availability

Not applicable.
